# Supplementary material for: Are carbon emissions trading and green financial instruments synergistic? -Comprehensive quantitative research based on content analysis
Source: PLoS One. 2024 Mar 7;19(3):e0298601. doi: 10.1371/journal.pone.0298601 (PMC10919601; doi:10.1371/journal.pone.0298601)
Supplement: S1 Data — (ZIP) [file pone.0298601.s001.zip › Data/地级市Z_F工作报告.zip!/║╙▒▒/║¬╡a╩╨/└┤╫╘╣1⁄2╓┌║┼í╛┬φ┐╦╩2╛▌═°í┐_2018.docx]

各位代表：

现在，我代表市人民政府，向大会报告工作，请予审议，并请各位市政协委员和列席人员提出意见。

一、2017年工作回顾

2017年是本届政府的开局之年，我们在省委、省政府和中共邯郸市委的坚强领导下，在市人大、市政协的监督支持下，坚持以习近平新时代中国特色社会主义思想为指导，深入学习贯彻党的十九大精神，紧紧围绕建设宜居宜业宜游富强邯郸、美丽邯郸的战略目标，攻坚克难，砥砺奋进，较好完成了市十五届人大一次会议确定的目标任务，办成了许多事关长远的大事难事，为新征程上再出发奠定了坚实基础。特别是我市荣获全国文明城市、全国社会治安综合治理优秀市等多项国家级荣誉，实现了邯郸人民多年的夙愿。

一年来，我们把稳定增长作为首要任务，致力稳中求进、提质增效，综合实力得到新提升。以项目建设为发展生命线，坚持项目“三集中”，创新“四色”管理法，争列省重点项目33个，数量居全省第一，省、市重点项目均超额完成全年投资计划，格力中原智能装备基础件产业基地、中船重工718所特种气体、峰峰鑫宝新材料、永年金力锂电池隔膜、邱县科莱电梯等一批产业项目开工建设或竣工投产。全市生产总值完成3666.3亿元，增长7.1%；全部财政收入367.7亿元，增长12.8%；一般公共预算收入220.1亿元，增长7.6%；规模以上工业增加值完成1370.3亿元、增长5%，实现利润285.1亿元、增长55.1%；固定资产投资3977.9亿元，增长5.6%；社会消费品零售总额完成1674.2亿元，增长11%；实际利用外资10.9亿美元，增长6.9%；城镇、农村居民人均可支配收入分别达28774元、13151元，均增长8.2%。

一年来，我们把治理污染作为头等大事，致力重拳出击、日日攻坚，生态环境有了新改善。扎实开展蓝天、碧水、净土三大行动，以前所未有的决心和力度推进生态环境建设。坚持科学治霾、精准治霾、协同治霾、铁腕治霾，打响大气污染综合治理生死战，制定实施“1+29”方案，深入开展秋冬季攻坚行动，统筹压煤、优企、控车、抑尘、治矿、增绿。完成气代煤、电代煤改造14.2万户，整治“散乱污”企业29201家，永年标准件、鸡泽铸造产业规范提升全面完成。预计煤炭消费量比上年削减165万吨以上，单位生产总值能耗下降5%以上。实施钢铁焦化行业污染整治行动，建立了企业无组织排放全口径清单，强化涉气企业挥发性有机物治理。“一控两防三圈四保”货车专项整治扎实开展，大力实施“汽运”改“铁运”，取缔非法加油站点1022家。主城区电动公交车达到1248辆，我市成为全省首个主城区公交车全部电动化城市。强力推进“五个三”综合整治，累计拆违近1000万平方米，整治经验在全省推广，荣获全省“一区三边”集中整治工作先进市。市县环保机构监测监察执法垂直管理体制改革全面完成，建立了大气网格化监管体系，在绕城高速公路内布设196个监测点位，全市243个乡镇均安装小型空气监测站，组建了2000多人的市民环保监督团。持续加大量化问责、执法监管力度，错峰生产、错峰运输、应急响应工作有序推进。去年，全市平均降尘量由1月份的13.3吨/平方公里，降到了12月份的4.2吨/平方公里；全市PM平均浓度为86微克/立方米，较2013年下降37.6%，超额完成大气“国十条”确定的目标任务。去年四季度，PM平均浓度同比下降26.5%，秋冬季空气质量为五年来最好，群众感受度明显好于往年。修复露天矿山30处，实现两年任务一年完成。划定生态保护红线，完成造林绿化79.1万亩，占年任务的120%。加强土壤和水污染治理，大力整治入河排污口、纳污坑塘，全面建立河长制，完成5条黑臭水体整治任务，地下水超采综合治理全省领先，我市列入全国首批水生态文明城市。

一年来，我们把结构调整作为主攻方向，致力加快转型、提档升级，发展方式实现新转变。产业结构日趋合理。三次产业比重由2016年的12.4：47.6：40，调整为11.1：48.6：40.3。去产能任务提前超额完成。压减炼铁产能484万吨、炼钢产能354万吨，邯郸纵横和武安东山、广耀、运丰等4家钢铁企业整体退出，压减煤炭产能187万吨、焦炭产能350万吨、火电装机规模30.7万千瓦，也超额完成了省“6643”工程下达我市任务。传统产业改造加快。永洋特钢产业园一期投产，冀南钢铁退城进园项目开工建设，技改投资增长6.8%，规模以上轻工业增加值增长19.5%。战略性新兴产业增长提速。高新技术产业增加值增长30.9%，居全省第一。装备制造业增加值增长45.3%，对规模以上工业增长贡献率达132.6%。新增规模以上工业企业199家，新增中国驰名商标3件、省名牌产品71件。现代服务业繁荣活跃。服务业增加值增长11.1%，对经济增长贡献率高于二产36.1个百分点。在涉县、峰峰矿区成功举办两届市旅发大会，创造了“旅发速度”和“旅发精神”，我市列入全省全域旅游示范区创建单位，广府古城被评为国家水利风景区，永年洼列入国家湿地公园，全市接待游客5859.8万人次，旅游总收入638.5亿元，分别增长23.6%和30.4%。现代农业稳步发展。粮食总产552.6万吨，农业产业化经营率预计达68%，邯郸国家农业科技园区通过验收，滏东现代农业产业园列入国家级产业园创建单位，肥乡区被确认为国家有机产品认证示范创建区，邱县荣获全国食品工业强县，鸡泽辣椒入选中国特色农产品优势区，新增省级农业科技园区3家、现代农业园区8家，新增国家地理标志保护产品和地理标志商标4件。

一年来，我们把改革创新作为强大动力，致力重点突破、活跃全局，经济发展焕发新活力。重点领域改革成效明显。完成年度改革任务344项。持续深化“放管服”改革，市、县两级全部组建了行政审批局，取消市直单位公章2894枚，推行群众和企业办事“一趟清”“不见面”改革，整合并设立“961890”群众服务热线，“双随机一公开”监管实现全覆盖，营商环境明显改善，全市新设市场主体9.2万户。城发、建投、交投、水投、市政、文体旅等六大投融资集团改革取得突破性进展，城市执法体制改革、“三供一业”分离移交全部完成。开展六大专项行动，全面优化金融生态环境，新增挂牌企业54家、村镇银行3家，农村信用社改制步伐加快，大名农商行挂牌开业。生育保险和职工基本医疗保险合并试点、城市公立医院综合改革、农村改革、供销社综合改革等各项改革深入推进。广泛开展扶工助企活动，万名干部大走访，帮助企业解难题。民营经济增加值增长7.3%。对外开放不断扩大。积极融入京津冀协同发展，成功举办第二届世界冀商大会、京津冀县域经济发展研讨会等重大活动，累计签约项目746个，引进京津合作项目226个，中文在线、中国建材凯盛集团等一批知名企业落户邯郸。复兴经济开发区获批省级开发区，20家入统省级以上开发区主营业务收入增长29.4%。创新驱动步伐加快。北京市科学技术研究院邯郸分院挂牌成立，我市与北京大学马克思主义学院签订全面战略合作协议，列入全省首批大学生创新创业试点市。新增高新技术企业86家、科技型中小企业1591家，新增院士工作站3家、省级科技孵化器4家、省级以上众创空间5家，荣获国家科技进步奖4项。

一年来，我们把新型城镇化作为有力引擎，致力夯实基础、补齐短板，城乡面貌发生新改观。坚持城市建设、城市管理、城市经济“三城齐抓”，生产、生活、生态“三生融合”，全面加快新型城镇化进程。东区开发建设提速，市科技中心、市客运中心站投入使用。主城区新建改建道路28条、小街巷20条，着力加强供暖、供气等公共服务保障，城市功能不断完善。发动全民抓创城，推进洁净城市建设，从市政设施、老旧小区、集贸市场到窗口服务、交通秩序、市民素质得到全面提升，城市面貌发生了翻天覆地的变化，赢得了“全国文明城市”这项含金量最高的荣誉，临漳、广平、鸡泽、成安、魏县、曲周、涉县等7个县城荣获省级文明县城。我市列入全国装配式建筑示范城市，邯银大厦荣获国家优质工程奖，主城区老旧小区改造提升和紫山工矿废弃地生态修复项目荣获省人居环境范例奖。邯郸机场新增5条航线，旅客吞吐量达68.4万人次，位居全省支线机场第一。引黄入冀补淀工程顺利通水，南水北调20个配套水厂全部完成水源切换，380多万群众喝上优质长江水。县城及美丽乡村建设扎实推进，曲周、魏县荣获国家园林县城，肥乡区天台山镇列入全国特色小城镇，峰峰矿区响堂水镇荣获“2017CCTV中国十大最美乡村”。全市常住人口城镇化率达到55.31%，比上年提高1.78个百分点。

一年来，我们把改善民生作为根本目的，致力以人为本、共建共享，各项社会事业取得新成效。财政用于民生支出374亿元，82件民生实事全部完成，省定286项灾后重建工程全部完成年度任务，均达到或超过灾前水平。突出精准扶贫、精准脱贫、精准防贫，7.9万贫困人口稳定脱贫。城镇新增就业16.9万人，下岗失业人员再就业4.38万人，城乡居民基本医保制度实现统一，异地就医实现跨省直接结算。大力解决房地产遗留问题，盘活安居东城、万达公馆等开工难、办证难、征迁难项目353个，解决了困扰15.1万户群众多年的烦心事。棚户区开工改造10542套、基本建成5221套，公租房基本建成3671套，改造农村危房2181户。教育、文化、卫生、体育等各项社会事业协调发展，建成中小学35所、幼儿园60所，各县（市、区）全部通过义务教育发展基本均衡县（市、区）认定，邺城遗址公园列入国家考古遗址公园立项名单，电视剧《太行山上》入选中宣部“五个一工程”奖，我市“健康小屋”典型经验受到国务院第四次大督查通报表扬，连续三年荣获全省医改工作先进市。高度重视信访稳定，依法打击和处置非法集资，认真落实军队退役人员政策，强化食品药品安全监管，安全生产形势总体平稳，平安邯郸建设扎实推进，全面开展严打、严治、严防、严控，刑事发案率下降12.8%，社会大局和谐稳定，人民群众获得感、幸福感、安全感不断增强。国防动员、双拥共建和优抚安置工作不断加强。民族宗教、新闻出版、广播电视、外事侨务、邮政通信、史志档案、人民防空、气象地震、援藏援疆、妇女儿童、老龄、残疾人等事业都取得了新进步。

一年来，我们把自身建设作为重要保障，致力转变作风、提升效能，政府执行力和公信力得到新增强。坚决落实全面从严治党的战略部署，推进“两学一做”学习教育常态化制度化，认真落实中央八项规定及实施细则精神，驰而不息纠正“四风”，深化机关作风整顿，行政监察、审计监督、责任追究和反腐倡廉工作力度不断加大。

坚持“四个干”、“六个点”、微信群、网格化等工作机制，推行扁平化、信息化、精细化管理，充分调动了政府系统抓落实的自觉性和积极性。坚持依法科学民主行政，市政府常务会议会前学法制度进一步完善，行政执法三项制度试点工作全面铺开，政府智库启动建设，邀请市民代表旁听市政府常务会议，重大行政决策制度更加完善。自觉接受市人大及其常委会的监督、市政协民主监督，办理人大代表建议470件、政协提案502件，按时办复率均为100%。

各位代表！过去一年，我市正处在爬陡坡、过深坎的关键时期，稳增长、促改革、调结构、治污染、惠民生、防风险任务异常艰巨，取得成绩来之不易。这是以习近平新时代中国特色社会主义思想领航定向的结果，是省委、省政府和市委坚强领导的结果，是市人大、市政协和社会各界鼎力支持的结果，是全市广大干部群众团结奋斗的结果。在此，我代表市人民政府，向全市人民，向人大代表、政协委员，向各民主党派、工商联、无党派人士和人民团体，向各位老领导、老同志，向驻邯人民解放军、武警官兵和政法干警，向中直、省直驻邯各单位，向所有关心支持邯郸发展的同志们、朋友们，致以崇高的敬意和衷心的感谢！

在肯定成绩的同时，我们也清醒地认识到，我市发展不平衡不充分的问题仍然存在，主要是：发展质量和效益还不高，产业结构重、资源消耗大；科技创新能力较弱，发展动能转换还不快；城乡区域发展不够协调，城镇发展质量还不高，城市经济、县域经济发展不够充分；环境污染问题仍很突出，大气、水、土壤治理任务十分繁重；金融领域、社会稳定风险不容忽视，一些新的矛盾和问题凸显；民生和社会事业还有不少短板，就业、教育、医疗、养老等方面还不能很好满足人民群众需要；营商环境仍需改善，政府部门服务质量和效率有待进一步提高，一些工作人员不担当不作为问题不同程度存在。同时，PM2.5平均浓度同比不降反升，出口总值负增长，没有达到预期目标。对此，我们一定坚持问题导向，强化责任担当，下大力予以破解。

各位代表！历史车轮滚滚向前，时代潮流浩浩荡荡。只要我们始终保持革命精神、革命斗志，始终保持永不懈怠的精神状态和一往无前的奋斗姿态，勇于变革，勇于创新，就一定能够战胜前进道路上的任何困难和挑战，开创新时代政府各项工作的新局面！

二、2018年主要工作

今年是贯彻落实党的十九大精神的开局之年，是改革开放40周年，是决胜全面建成小康社会、实施“十三五”规划承上启下的关键一年。做好今年各项工作，意义重大、影响深远。

今年政府工作的总体要求是：以习近平新时代中国特色社会主义思想为指导，全面贯彻落实党的十九大精神，按照省委九届五次、六次全会和市委九届三次全会工作部署，坚持稳中求进工作总基调，把握底定超干工作总要求，坚持新发展理念，紧扣我国社会主要矛盾变化，按照高质量发展的要求，统筹推进“五位一体”总体布局，协调推进“四个全面”战略布局，以供给侧结构性改革为主线，坚持创新竞进、协同融合、改革开放、转型升级、提质增效、改善民生、优化环境，全面强化项目支撑、创新引领、开放带动三大动力，统筹推进城市提升、县城突破、乡村振兴三大战略，坚决打好防范化解重大风险、精准脱贫、污染防治三大攻坚战，有力推动经济发展质量变革、效率变革、动力变革，加强和改善民生，维护安全稳定大局，加快建设京津冀联动中原的区域中心城市，奋力开启新时代全面建设富强邯郸美丽邯郸新征程。

全市经济社会发展的主要预期目标是：全市生产总值增长6.5%左右，一般公共预算收入增长7%，规模以上工业增加值增长5%左右，固定资产投资增长5%左右，社会消费品零售总额增长11%以上，实际利用外资增长5%，出口总值增长5%，城镇、农村居民人均可支配收入分别增长8%和8%以上，居民消费价格涨幅控制在3%左右，城镇登记失业率控制在4%以内，单位生产总值能耗、PM2.5平均浓度下降率和化学需氧量、二氧化硫、氨氮、氮氧化物等主要污染物减排完成省定任务。

实现上述目标，做好今年工作，必须把高质量发展这一根本要求贯穿经济社会发展各领域、全过程，以高质量发展引领邯郸转型升级、绿色发展、跨越提升。重点在十个方面取得突破。

（一）推动经济平稳增长，夯实高质量发展基础。充分发挥投资关键作用、消费基础作用、出口带动作用、实体经济支撑作用，保持稳中有进、稳中向好的发展态势，确保换挡不失速、量增质更优。以有效投资提质扩量。坚持项目建设“三集中”机制，突出抓好46个省重点项目、310个市重点项目，完成年度投资800亿元。优化政府投资方向，实施474个市县政府投资项目，完成年度投资331亿元。加大重大谋划项目推进力度，建立谋划储备项目库，动态存储项目1000个以上。强化“四色”管理，制定投入与产出强度标准，集约节约高效利用土地。更加注重绿色投资，坚决杜绝高消耗、高污染、高排放项目，以高质量投资优化供给结构。加快太行山高速公路邯郸段、西部旅游路网、邯郸机场三期等项目建设，积极推进京石邯城际铁路、聊邯长客专、城市轨道交通等项目前期工作，全面提升重大基础设施保障水平。激发民间投资活力，引导社会资本参与重点领域建设，努力形成多元化、可持续的资金投入机制。以消费升级激活需求。实施消费促进、电子商务进农村进社区、商贸物流配送体系建设、实体商业转型升级四大专项行动，加快发展文体旅游、教育培训、家政服务、健康养老等服务性消费，培育信息、绿色、时尚等新兴消费，扩大网络消费规模。加大4G网络深度覆盖力度，启动5G网络规划建设，优化信息消费环境。以扩大外贸增加动力。实施外贸三年攻坚提升计划，出台出口鼓励政策，及时足额退税，积极稳定外贸出口。加快外贸转型升级示范基地、贸易平台、国际营销网络建设，大力发展跨境电商，建设市级对外开放综合服务平台，支持永年标准件市场争创“市场采购贸易试点”，加快邯郸国际陆港、武安保税物流中心、青岛保税港区邯郸（鸡泽）功能区建设运营，打造8个县域特色产业外贸基地。坚持投资与贸易并重，鼓励钢铁、水泥等优势企业走出去，参与“一带一路”沿线地区建设。以服务实体做强支撑。大力发展实体经济，筑牢现代化经济体系的坚实基础。推动资源要素向实体经济集聚、政策措施向实体经济倾斜、工作力量向实体经济加强，扎实开展以创新创业、服务发展服务民生为主要内容的“双创双服”活动，全面落实重点企业联系服务“五个一”机制，深入推进合力扶工助企，送政策、解难题、促发展。开展经常性、制度化、专业型银企对接活动，争取引进5家金融机构，实施企业上市三年行动计划，年内力争2家企业上市，争取石交所在我市开展可转债试点，提高企业直接融资比例。支持邯郸银行邢台分行设立开业。开展小微企业产业园区建设试点，加快推进个转企、小升规，新增规模以上工业企业100家。

（二）深化供给侧结构性改革，提升高质量发展水平。坚持质量第一、效益优先，坚决去、主动调、加快转，做到“长大于消”“长优于消”，着力建设现代化经济体系。化解过剩产能倒逼转型升级。坚决落实省去产能三年行动计划，严格执行环保、能耗、水耗、质量、技术、安全等六类标准和规划、土地、社保、税收四个“杠杠”，用好绿色发展评估办法，力争压减炼铁产能86万吨，完成压减煤炭产能115万吨、焦炭产能300万吨以上、火电装机规模25.6万千瓦。加大“僵尸企业”处置力度，大力破除无效供给，年内2家企业出清。改造传统产业助推转型升级。制定传统产业转型发展三年行动计划，深入实施“千项技改工程”，三年内完成技改投资2000亿元。加快钢铁产业减量重组，开工建设武安太行、峰峰华信钢铁退城进园项目。推进信息化与工业化深度融合，深化“互联网+先进制造业”，培育10个省级智能工厂和数字化车间，积极创建“中国制造2025”国家级示范区。认真落实促进工业设计发展的政策措施，建设市级工业设计创新中心，培育10家工业设计示范企业，打造“设计+”产业链，提升品牌影响力和产品附加值。培育战略性新兴产业带动转型升级。落实省战略性新兴产业发展三年行动计划，推进百项高技术产业示范项目，重点培育新材料、新能源汽车、高端装备制造、绿色低碳、储能、氢能等战略性新兴产业，优先发展“大智移云”产业，抓好格力中原智能装备基础件产业基地、中化国际锂电池负极材料、中国电子健康医疗大数据中心与产业园等一批大项目，高新技术产业增加值增长15%以上。积极培育数字经济、共享经济、现代供应链等新业态新模式，努力形成新的经济增长点。认真落实《推进军民深度融合发展的实施意见》，深化与涉军单位战略合作，设立产业发展基金，重点抓好中船重工（邯郸）军民融合产业示范区建设，年内军民融合企业达到50家。做强现代服务业引领转型升级。繁荣商贸物流。推进主城区老旧市场整合外迁，邯郸华耀城、浙江商贸城、美食林环球中心等项目尽快竣工投用。推动线上线下融合发展，加快冀南（永年）快递产业园等项目建设，积极创建全国电子商务示范城市。激活全域旅游。加快赵王城、邺城、大名府、广府四大古城旅游区开发，建设武安东太行、涉县红河谷、峰峰响堂山、磁县溢泉湖等十大旅游片区，精心筹办第三届市旅游产业发展大会暨第十四届国际太极拳运动大会，旅游总收入增长25%以上，确保涉县、武安通过国家全域旅游示范区创建验收。建设文化强市。实施文化产业振兴“双百工程”，加强与中文在线、深圳华强集团的战略合作，推进邯郸文化IP化系统开发工程，启动新媒体产业园建设，中华成语文化博览园投入运营。积极促进文化与旅游、科技、金融等融合发展，打造“文化+”新业态。培育会展经济、节能服务、信息技术、金融保险等现代服务业，服务业增加值增长11%以上。严格质量标准促进转型升级。牢树大质量观、大标准观，落实《实施质量品牌战略提升计划》，推动标准、质量、品牌、信誉联动提升。支持企业参与制定修订国际、国家、行业标准，年内主导或参与制定各类标准14项。组织开展制造业、消费品、服务业等质量提升十大专项行动，新建省级以上质检中心2家，鼓励引导企业争创中国质量奖、中国工业大奖。挖掘打造一批知名品牌和“百年老店”，新增中国驰名商标3件、省名优产品80件、省服务品牌15个。整合信用信息，实现信息共享，健全守信联合激励和失信联合惩戒机制，引导市场主体以信誉赢得市场。
